# Poor prognostic implications of myelodysplasia-related mutations in both older and younger patients with *de novo* AML

**DOI:** 10.1038/s41408-022-00774-7

**Published:** 2023-01-04

**Authors:** Xavier Cheng-Hong Tsai, Kuo-Jui Sun, Min-Yen Lo, Feng-Ming Tien, Yuan-Yeh Kuo, Mei-Hsuan Tseng, Yen-Ling Peng, Yi-Kuang Chuang, Bor-Sheng Ko, Jih-Luh Tang, Hsun-I Sun, Ming-Chih Liu, Chia-Wen Liu, Chien-Chin Lin, Ming Yao, Wen-Chien Chou, Hsin-An Hou, Hwei-Fang Tien

**Affiliations:** 1 Department of Medical Education and Research, National Taiwan University Hospital Yunlin Branch, Yunlin, Taiwan; 2 Division of Hematology, Department of Internal Medicine, National Taiwan University Hospital, Taipei, Taiwan; 3 Department of Hematological Oncology, National Taiwan University Cancer Center, National Taiwan University Hospital, Taipei, Taiwan; 4 Department of Laboratory Medicine, National Taiwan University Hospital, Taipei, Taiwan; 5 Division of Hematology, Department of Internal Medicine, National Taiwan University Hospital Yunlin Branch, Yunlin, Taiwan; 6 Tai-Chen Cell Therapy Center, National Taiwan University, Taipei, Taiwan; 7 Department of Pathology, National Taiwan University Hospital, Taipei, Taiwan; 8 Department of Internal Medicine, Far-Eastern Memorial Hospital, New Taipei City, Taiwan

**Keywords:** Cancer epigenetics, Cancer genomics

## Abstract

A set of myelodysplasia-related (MDS-R) gene mutations are incorporated into the 2022 European LeukemiaNet risk classification as adverse genetic factors for acute myeloid leukemia (AML) based on their poor prognostic impact on older patients. The impact of these mutations on younger patients (age < 60 years) remains elusive. In the study of 1213 patients with *de novo* non-M3 AML, we identified MDS-R mutations in 32.7% of the total cohort, 44.9% of older patients and 23.4% of younger patients. The patients with MDS-R mutations had a significantly lower complete remission rate in both younger and older age groups. With a median follow-up of 9.2 years, the MDS-R group experienced shorter overall survival (*P* = 0.034 for older and 0.035 for younger patients) and event-free survival (*P* = 0.004 for older and 0.042 for younger patients). Furthermore, patients with MDS-R mutations more frequently harbored measurable residual disease that was detectable using next generation sequencing at morphological CR than those without MDS-R mutations. Allogeneic hematopoietic stem cell transplantation (allo-HSCT) might ameliorate the negative impact of MDS-R mutations. In summary, AML patients with MDS-R mutations have significantly poorer outcomes regardless of age. More intensive treatment, such as allo-HSCT and/or novel therapies, is warranted for AML patients with MDS-R mutations.

## Introduction

Acute myeloid leukemia (AML) is a biologically and clinically heterogeneous hematologic malignancy that is characterized by abnormal proliferation and differentiation of clonal hematopoietic stem/progenitor cells [1]. Because of the remarkably complex diversity of this disease, proper risk stratification is the cornerstone to maximize treatment efficacy and minimize treatment-related toxicities.

In addition to patient-related risk factors [2, 3], there have been numerous efforts to explore disease-associated prognostic factors for AML patients, including the most widely utilized 2017 European LeukemiaNet (ELN) recommendation [4], incorporating a variety of cytogenetic and gene mutation profiles in the risk stratification. Researchers have also sought to refine the 2017 ELN recommendation by incorporating additional prognostic markers consisting of aberrations in coding [5] and noncoding [6] genes. Recently, mutations in a set of eight genes, including *ASXL1*, *BCOR*, *EZH2*, *SF3B1, SRSF2*, *STAG2*, *U2AF1*, and *ZRSR2* mutations, were categorized as secondary AML (sAML)-type mutations owing to their strong association with secondary AML that transformed from myelodysplastic syndrome (MDS) or chronic myelomonocytic leukemia (CMMoL) [7]. Despite the possibility of the presence of an unrecognized antecedent myelodysplasia before AML diagnosis in patients with sAML-type mutations, such mutations can be detected in more than 30% of patients with rigorously clinically defined *de novo* AML, and have been shown to confer a negative prognostic impact on elderly patients [7, 8]. Recently, AML harboring a panel of nine mutations encompassing the eight sAML-type mutations and *RUNX1* mutation have been categorized as AML with myelodysplasia-related (MDS-R) gene mutations in the 2022 International Consensus Classification (ICC) [9], and the adverse-risk group in the 2022 ELN risk classification [10]. However, the impact of MDS-R mutations on the survival of younger patients with clinically confirmed *de novo* AML remains to be investigated. Furthermore, little is known about the differences in the distribution of MDS-R mutations and their prognostic impact between different age groups; either is the association between MDS-R mutations and other genotypes. In this study, we aimed to explore the association of MDS-R mutations with clinic-biological features, molecular genetic alterations, and prognostic relevance in both older and younger age groups in a large cohort of *de novo* AML patients. The findings of this study may not only validate the current risk stratification system but also provide insight for further refinement.

## Materials and methods

### Patients and samples

From April 1994 to January 2021, 1464 consecutive adult patients were newly diagnosed as having *de novo* AML and treated at the National Taiwan University Hospital (NTUH). Patients with antecedent hematological diseases, a cytopenia history, family history of myeloid neoplasms, or exposure to leukemogenic therapy were not included. Patients with FAB M3 AML (*n* = 135) and those without adequate cryopreserved diagnostic bone marrow (BM) specimens for molecular analyses or who did not provide informed consent (*n* = 116) were excluded (Supplementary Fig. 1). Finally, a total of 1,213 patients with complete clinical, molecular, and laboratory data were enrolled in this study. AML was diagnosed according to the 2016 World Health Organization (WHO) classification [11] and the 2022 ICC [9].

To evaluate the association between measurable residual disease (MRD) clearance and MDS-R mutations, a subgroup of 291 patients who had paired BM samples obtained serially at diagnosis, complete remission (CR), and after the first consolidation chemotherapy and had detectable gene mutations other than *DNMT3A*, *TET2*, and *ASXL1* at diagnosis, as described previously [12], were enrolled for MRD monitoring using next generation sequencing (NGS). This retrospective study was approved by the NTUH Research Ethics Committee, and written informed consent was obtained from all participants in accordance with the Declaration of Helsinki (Approval number: 201709072RINC).

### Treatment

Patients eligible for standard intensive chemotherapy received standard 3+7 induction chemotherapy (idarubicin 12 mg/m^2^/day on days 1–3 and cytarabine 100 mg/m^2^/day on days 1–7; 2+5 was permitted for older patients) and 2–4 courses of postremission chemotherapy with high-dose cytarabine (2000 mg/m^2^ twice per day on days 1–4), with or without anthracycline [12]. Patients receiving standard intensive chemotherapy were included in the survival analysis. Treatment response was evaluated according to the ELN recommendation [10]. The choice of allogeneic HSCT was based on molecular risk stratification, age, comorbidities, availability of donors, and response to induction treatment, as evaluated by morphological observation and multicolor flow cytometry examination, which is a routine test at our institute [12]. The median follow-up time of this cohort was 9.2 years.

### Molecular mutation analyses using next generation sequencing (NGS)

NGS was performed using the TruSight myeloid sequencing panel and HiSeq platform (Illumina, San Diego, CA) to evaluate mutations in 54 myeloid malignancy-related genes (Supplementary Table 1). Library preparation and sequencing were performed according to the manufacturer’s instructions. The median reading depth was 11,000×. We used COSMIC database version 86 [13], dbSNP version 151 [14], ClinVar [15], 1000 Genomes [16], PolyPhen-2 [17], and SIFT [18] to evaluate the consequence of every variant. The detailed variant analysis algorithm for diagnostic samples was described previously [19], with a minimum variant allele frequency of 5%. Because of an issue with sequencing sensitivity, *CEBPA* mutations and *FLT3*-ITD were evaluated using Sanger sequencing and fragment analysis, respectively [20, 21]. Cytogenetic analysis was performed as previously described [2]; the classification was performed according to refined Medical Research Council (MRC) criteria [22]. MRD monitoring using NGS was performed as previously described [12].

### Statistical analysis

Continuous variables and medians of distributions were compared using the Mann–Whitney U or Kruskal–Wallis test. The difference between discrete variables was compared using the chi-square test or Fisher’s exact test. Overall survival (OS) was defined from the date of initial diagnosis to the date of last follow-up or death from any cause, and event-free survival (EFS) was defined from the date of initial diagnosis to the date of treatment failure, hematologic relapse, or death from any cause, whichever occurred first [10]. Kaplan–Meier analysis was employed to calculate survival probabilities and the log-rank test was used to evaluate the statistical significance. The Cox proportional hazards model was applied for the multivariate regression analysis and to generate hazard ratios (HRs) and 95% confidence intervals (CIs). To accurately evaluate the effect of allo-HSCT at first CR, we used the median time from remission to allo-HSCT (0.31 years) for the landmark analysis. In multivariate analysis, allo-HSCT at first CR was considered a time-dependent variable. All statistical analyses were performed with R version 4.1.1 (https://cran.r-project.org/). A two-sided *P* value less than 0.05 was considered statistically significant.

## Results

### Distinct clinical and laboratory features of patients with MDS-R mutations in different age groups

Among the 1213 patients recruited, 528 (43.5%) were aged more than 60 years (older group), with a median age of 71 years (range 61–98). MDS-R mutations were detected in 32.7% of total cohort, 44.9% of older patients and 23.4% of younger patients (≤60 years). In both younger and older groups, patients with MDS-R mutations were significantly older (*P* = 0.018 and *P* = 0.003, respectively). In the younger group, MDS-R mutations were associated with a lower white blood cell (WBC) count (*P* < 0.001), lower peripheral blood blast count (*P* < 0.001), and lower lactate dehydrogenase (LDH) level (*P* < 0.001) at diagnosis, but there was no difference in the older group (Table 1).

**Table 1:** Comparison of clinical characteristics between AML patients with and without MDS-R mutations^e^.

Variables	Younger population			Older population		
	Without MDS-R mutations (*n* = 525, 76.6%)	With MDS-R mutations (*n* = 160, 23.4%)	*P* value	Without MDS-R mutations (*n* = 291, 55.1%)	With MDS-R mutations (*n* = 237, 44.9%)	*P* value
Sex^a^			0.365			<0.001
Male	279 (53.1%)	92 (57.5%)		154 (52.9%)	165 (69.6%)	
Female	246 (46.9%)	68 (42.5%)		137 (47.1 %)	72 (30.4%)	
Age (years)^b^	41.1 (15–60)	45.2 (15–60)	0.018	69.7 (61–98)	72.5 (61–92)	0.003
Lab data^b^						
WBCs (number/μL)	22,080 (80–405,650)	8,745 (390–374,500)	<0.001	10,390 (430–340,400)	9,730 (520–627,800)	0.988
Hb (g/dL)	8.2 (2.4–15.3)	7.8 (3.7–16.0)	0.063	8.2 (3.2–13.6)	8.3 (3.6–16.2)	0.347
Platelets (numbers×1000/μL)	49 (3–751)	50 (2–1017)	0.918	45 (3–424)	47 (3–463)	0.592
Peripheral blood blasts (numbers/μL)	8892 (0–373,198)	3008 (0–324,576)	<0.001	2620 (0–273,248)	1574 (0–456,724)	0.264
LDH (U/L)	764 (98–13,130)	561 (140–12,898)	<0.001	561 (129–15,000)	549 (96–13,893)	0.997
FAB^a^						
M0	11 (2.1%)	12 (7.5%)	0.001	7 (2.4%)	12 (5.1%)	0.103
M1	125 (23.8%)	39 (24.4%)	0.883	53 (18.2%)	39 (16.5%)	0.596
M2	208 (39.6%)	60 (37.5%)	0.631	130 (44.7%)	83 (35.0%)	0.025
M4	141 (26.9%)	33 (20.6%)	0.113	18 (6.2%)	87 (36.7%)	<0.001
M5	23 (4.4%)	7 (4.4%)	0.997	18 (6.2%)	11 (4.6%)	0.439
M6	17 (3.2%)	9 (5.6%)	0.167	8 (2.7%)	5 (2.1%)	0.637
2022 International Consensus Classification^a^						
AML with t(8;21)(q22;q22.1)/*RUNX1*::*RUNX1T1*	62 (11.8%)	19 (11.9%)	0.982	14 (4.8%)	2 (0.8%)	0.008
AML with inv(16)(p13.1q22) or t(16;16) (p13.1;q22)/*CBFB*::*MYH11*	38 (7.2%)	1 (0.6%)	0.002	8 (2.7%)	0 (0%)	0.010
AML with t(9;11)(p21.3;q23.3)/*MLLT3*::*KMT2A*	8 (1.5%)	1 (0.6%)	0.382	5 (1.7%)	2 (0.8%)	0.382
AML with other *KMT2A* rearrangements	19 (3.6%)	3 (1.9%)	0.273	3 (1.0%)	3 (1.3%)	0.800
AML with t(6;9)(p22.3;q34.1)/*DEK*::*NUP214*	7 (1.3%)	1 (0.6%)	0.465	1 (0.3%)	0 (0%)	0.366
AML with inv(3)(q21.3q26.2) or t(3;3)(q21.3;q26.2)/*GATA2*; *MECOM*(*EVI1*)	5 (1.0%)	6 (3.8%)	0.014	0 (0%)	1 (0.4%)	0.267
AML with other *MECOM* rearrangements	1 (0.2%)	1 (0.6%)	0.372	2 (0.7%)	0 (0%)	0.201
AML with other rare recurring translocations	20 (3.8%)	1 (0.6%)	0.041	5 (1.7%)	3 (1.3%)	0.672
AML with t(9;22)(q34.1;q11.2)/*BCR*::*ABL1*	3 (0.6%)	1 (0.6%)	0.938	2 (0.7%)	1 (0.4%)	0.687
AML with mutated *NPM1*	113 (21.5%)	5 (3.1%)	<0.001	102 (35.1%)	32 (13.5%)	<0.001
AML with in-frame bZIP *CEBPA* mutations	95 (18.1%)	10 (6.3%)	<0.001	16 (5.5%)	13 (5.5%)	0.995
AML with mutated *TP53*	20 (3.8%)	7 (4.4%)	0.748	49 (16.8%)	11 (4.6%)	<0.001
AML with myelodysplasia- related gene mutations	0 (0%)	104 (65.0%)	<0.001	0 (0%)	169 (71.3%)	<0.001
AML with myelodysplasia-related cytogenetic abnormalities	28 (5.3%)	0 (0%)	0.003	30 (10.3%)	0 (0%)	<0.001
AML not otherwise specified (NOS)	106 (20.2%)	0 (0%)	<0.001	54 (18.6%)	0 (0%)	<0.001
Cytogenetic-risk^a,c^			0.243			<0.001
Favorable	98 (18.8%)	21 (13.5%)		21 (7.4%)	2 (0.9%)	
Intermediate	358 (68.7%)	111 (71.2%)		179 (63.0%)	190 (81.9%)	
Unfavorable	65 (12.5%)	24 (15.4%)		84 (29.6%)	40 (17.2%)	
2017 ELN risk-stratification^a,f^			<0.001			<0.001
Favorable	250 (47.6%)	35 (21.9%)		105 (36.1%)	29 (12.2%)	
Intermediate	168 (32.0%)	28 (17.5%)		82 (28.2%)	39 (16.5%)	
Unfavorable	107 (20.4%)	97 (60.6%)		104 (35.7%)	169 (71.3%)	
Induction responses^a,d^						
CR	415 (83.8%)	106 (72.1%)	0.001	103 (68.2%)	51 (52.0%)	0.010
PR/Refractory	64 (12.9%)	35 (23.8%)	0.001	33 (21.9%)	40 (40.8%)	0.001
Induction death	16 (3.2%)	6 (4.1%)	0.619	15 (9.9%)	8 (8.2%)	0.637
HSCT at first CR^a^	125 (25.3%)	39 (26.5%)	0.755	22 (14.6%)	11 (11.2%)	0.447
Relapse^a^	176 (42.4%)	35 (33.0%)	0.079	49 (47.6%)	26 (51.0%)	0.691

*AML* acute myeloid leukemia, *CR* complete remission, *ELN* European LeukemiaNet, *FAB* French-American-British classification, *Hb* hemoglobin, *LDH* lactate dehydrogenase, *MDS-R* myelodysplasia-related, *PR* partial remission, *WBC* white blood cell.

^a^Number of patients (%).

^b^Median (range).

^c^According to the refined Medical Research Council criteria. Cytogenetics data were available for 1193 patients.

^d^In the younger population, 495 patients without MDS-R mutations and 147 patients with MDS-R mutations received standard chemotherapy; in the older population, 151 patients without MDS-R mutations and 98 patients with MDS-R mutations received standard chemotherapy.

^e^The percentage may not sum to 100 because of rounding.

^f^Since AML with MDS-R gene mutations belongs to the adverse category in the 2022 ELN risk classification, the comparison of the risk groups based on this classification between AML patients with and without MDS-R is not meaningful, and thus we used the 2017 ELN risk classification.

In both younger and older groups, patients with MDS-R mutations had a lower chance of being categorized into the group of AML with inv(16) (*P* = 0.002 and *P* = 0.010, respectively, Table 1), AML with *NPM1* mutation (both *P* < 0.001), AML with myelodysplasia-related cytogenetic abnormalities (*P* = 0.003 and *P* < 0.001, respectively), or AML not otherwise specified (both *P* < 0.001). In addition, younger patients with MDS-R mutations more frequently had AML with inv(3) or *t*(3;3) and less frequently had AML with other rare recurrent translocation or in-frame bZIP *CEBPA* mutations. Older patients with MDS-R mutations less commonly had AML with *t*(8;21) or AML with mutated *TP53*.

### Patterns of cytogenetic abnormalities and gene mutations among patients with MDS-R mutations in different age groups

**Fig. 1:**
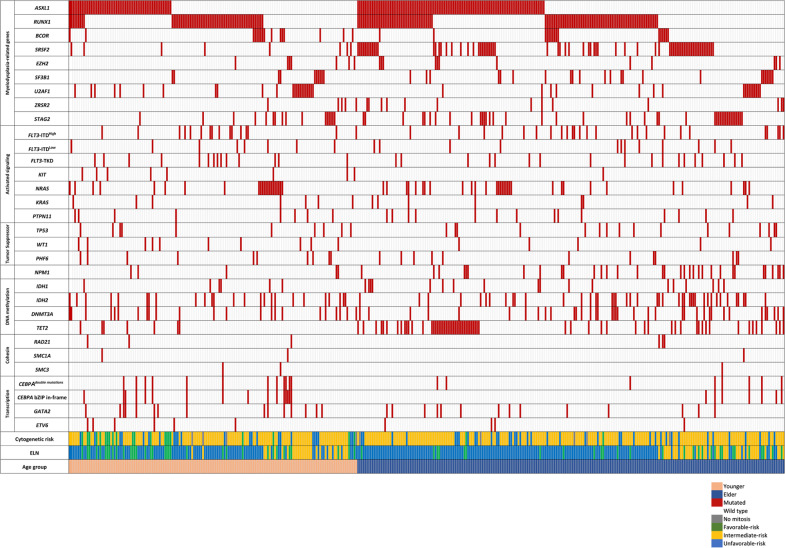
Comutation plot showing the complex interactions between MDS-R mutations and other mutations. Each column represents a patient. The left side of the plot represents the younger patient group and the right side represents the elder patient group (the last row).

We next investigated cytogenetic changes and gene mutations among patients with and without MDS-R mutations in different age groups. Regarding cytogenetic changes based on the refined Medical Research Council criteria [22], two-thirds of patients (69.3% of younger patients and 71.5% of older patients) showed intermediate-risk cytogenetic changes. Older patients carrying MDS-R mutations had a significantly lower frequency of unfavorable-risk cytogenetic changes (Table 1). Regarding gene mutation profiles, the most common gene mutation was *FLT3* mutations (26.7%), including *FLT3*-ITD (19.2%), *FLT3-*TKD (6.3%), and concomitant *FLT3*-ITD and *FLT3*-TKD (1.2%), followed by *NPM1* (21.2%) and *DNMT3A* mutations (17.8%). The mutation spectra of the patients with MDS-R mutations among the younger and older groups are shown in Fig. 1 and the comparison of the mutation pattern between the patients with and without MDS-R mutations in the two age groups is shown in Table 2. In the younger group, patients with MDS-R mutations had lower rates of *CEBPA* double mutations and *CEBPA* bZIP in-frame mutations but a higher rate of *IDH2* and *ETV6* mutations than those without MDS-R mutations. In the older group, patients with MDS-R mutations harbored fewer *KIT*, *TP53*, and *DNMT3A* mutations but more *TET2* mutations than those without these mutations. Furthermore, both younger and older patients with MDS-R mutations had fewer *NPM1* mutations but more *PHF6* mutations. Among the total cohort, 20.4% of patients had one MDS-R mutation and 12.4% had two or more MDS-R mutations. Older patients had significantly higher rates of one MDR-R mutation (older vs. younger: 23.3% vs. 18.1%, *P* = 0.026) as well as two or more MDR-R mutations (21.6% vs. 5.3%, *P* < 0.001) than younger ones. As expected, patients with MDS-R mutations were more frequently stratified into the unfavorable-risk group based on the 2017 ELN risk classification, regardless of a younger or older age status, since *ASXL1* and *RUNX1* mutations are assigned to the unfavorable-risk category by this classification.

**Table 2:** Comparison of gene mutations between AML patients with and without MDS-R mutations.

Categories	Variables	Total cohort (*N* = 1213)	Younger population			Older population		
			Without MDS-R mutations (*n* = 525, 76.6%)	With MDS-R mutations (*n* = 160, 23.4%)	*P* value	Without MDS-R mutations (*n* = 291, 55.1%)	With MDS-R mutations (*n* = 237, 44.9%)	*P* value
Activated signaling	*FLT3*-ITD^High a,b^	152 (12.5%)	76 (14.6%)	16 (10.0%)	0.146	35 (12.0%)	25 (10.5%)	0.594
	*FLT3*-ITD^Low a,b^	93 (7.7%)	48 (9.2%)	8 (5.0%)	0.094	25 (8.6%)	12 (5.1%)	0.114
	*FLT3*-TKD	91 (7.5%)	37 (7.0%)	14 (8.8%)	0.492	22 (7.6%)	18 (7.6%)	>0.999
	*KIT*	57 (4.7%)	34 (6.5%)	8 (5.0%)	0.576	14 (4.8%)	1 (0.4%)	0.002
	*NRAS*	169 (13.9%)	76 (14.5%)	22 (13.8%)	0.898	39 (13.4%)	32 (13.5%)	>0.999
	*KRAS*	51 (4.2%)	28 (5.3%)	7 (4.4%)	0.837	8 (2.7%)	8 (3.4%)	0.800
	*PTPN11*	69 (5.7%)	30 (5.7%)	8 (5.0%)	0.845	17 (5.8%)	14 (5.9%)	>0.999
Tumor suppressor	*TP53*	111 (9.2%)	25 (4.8%)	10 (6.3%)	0.420	60 (20.6%)	16 (6.8%)	<0.001
	*WTI*	79 (6.5%)	50 (9.5%)	10 (6.3%)	0.263	14 (4.8%)	5 (2.1%)	0.106
	*PHF6*	34 (2.8%)	8 (1.5%)	10 (6.3%)	0.003	4 (1.4%)	12 (5.1%)	0.020
	*NPM1*	257 (21.2%)	116 (22.1%)	5 (3.1%)	<0.001	103 (35.4%)	33 (13.9%)	<0.001
DNA methylation	*IDH1*	77 (6.3%)	31 (5.9%)	6 (3.8%)	0.423	22 (7.6%)	18 (7.6%)	>0.999
	*IDH2*	148 (12.2%)	38 (7.2%)	25 (15.6%)	0.003	42 (14.4%)	43 (18.1%)	0.284
	*DNMT3A*	216 (17.8%)	83 (15.8%)	23 (14.4%)	0.709	73 (25.2%)	37 (15.6%)	0.007
	*TET2*	162 (13.4%)	40 (7.6%)	7 (4.4%)	0.210	51 (17.5%)	64 (27.0%)	0.011
Cohesin complex genes	*STAG2*	57 (4.7%)	0 (0%)	16 (10.0%)	<0.001	0 (0%)	41 (17.3%)	<0.001
	*RAD21*	25 (2.1%)	17 (3.2%)	3 (1.9%)	0.591	2 (0.7%)	3 (1.3%)	0.661
	*SMC1A*	18 (1.5%)	11 (2.1%)	2 (1.3%)	0.743	4 (1.4%)	1 (0.4%)	0.257
	*SMC3*	9 (0.7%)	3 (0.6%)	2 (1.3%)	0.332	3 (1.0%)	1 (0.4%)	0.631
Transcription factor	*CEBPA*^double mutations^	103 (8.5%)	73 (13.9%)	12 (7.5%)	0.039	10 (3.4%)	8 (3.4%)	>0.999
	*CEBPA* bZIP in-frame	121 (10.0%)	87 (16.6%)	16 (10.0%)	0.042	11 (3.8%)	7 (3.0%)	0.603
	*GATA2*	84 (6.9%)	41 (7.8%)	15 (9.4%)	0.513	14 (4.8%)	14 (5.9%)	0.697
	*ETV6*	18 (1.5%)	6 (1.1%)	6 (3.8%)	0.039	2 (0.7%)	4 (1.7%)	0.416
	*RUNX1*	165 (13.6%)	0 (0%)	60 (37.5%)	<0.001	0 (0%)	105 (44.3%)	<0.001
Chromatin modifiers	*ASXL1*	161 (13.3%)	0 (0%)	57 (35.6%)	<0.001	0 (0%)	104 (43.9%)	<0.001
	*BOCR*	32 (2.6%)	0 (0%)	16 (10.0%)	<0.001	0 (0%)	16 (6.8%)	<0.001
	*EZH2*	19 (1.6%)	0 (0%)	7 (4.4%)	<0.001	0 (0%)	12 (5.1%)	<0.001
Spliceosome complex genes	*SF3B1*	33 (2.7%)	0 (0%)	10 (6.3%)	<0.001	0 (0%)	23 (9.7%)	<0.001
	*SRSF2*	80 (6.6%)	0 (0%)	8 (5.0%)	<0.001	0 (0%)	72 (30.4%)	<0.001
	*U2AF1*	41 (3.4%)	0 (0%)	23 (14.4%)	<0.001	0 (0%)	18 (7.6%)	<0.001
	*ZRSR2*	18 (1.5%)	0 (0%)	5 (3.1%)	0.001	0 (0%)	13 (5.5%)	<0.001

^a^Three patients with *FLT3*-ITD did not have allelic ratio data. All three young patients had no MDS-R mutations.

^b^Defined as *FLT3* mutated/wild-type allelic ratio ≥ 0.5.

### Negative prognostic impact of MDS-R mutations in both age groups

In total, 642 (93.7%) younger patients and 249 (47.2%) older patients received standard intensive chemotherapy. Among these patients, those with MDS-R mutations had significantly lower CR rates (83.8% vs 72.1%, *P* = 0.001 for the younger patients and 68.2% vs 52.0%, *P* = 0.010 for the older patients), but relapse rates were comparable (Table 1).

**Fig. 2:**
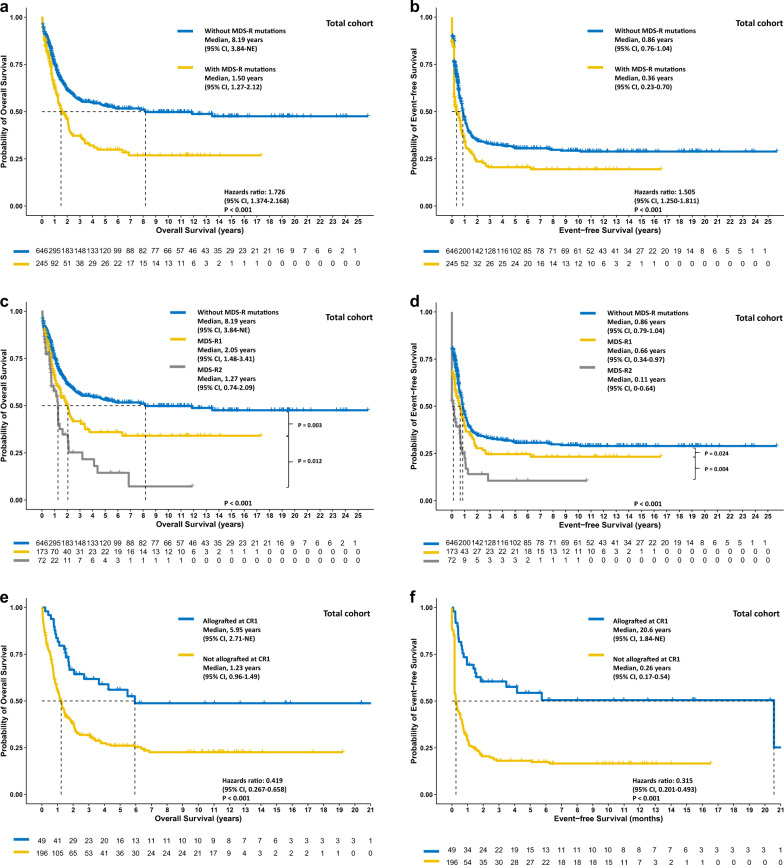
The Kaplan‒Meier survival curves for the total cohort. OS (**a**) and EFS (**b**) stratified by the status of MDS-R mutations, the MDS-R mutation burdens (**c** and **d**, respectively), and treatment with or without allogeneic transplantation at first remission (**e** and **f**, respectively). Allogeneic transplantation may overcome the negative prognostic impact of MDS-R mutations. MDS-R1, with one MDS-R mutation; MDS-R2, with 2 or more MDS-R mutations.

**Fig. 3:**
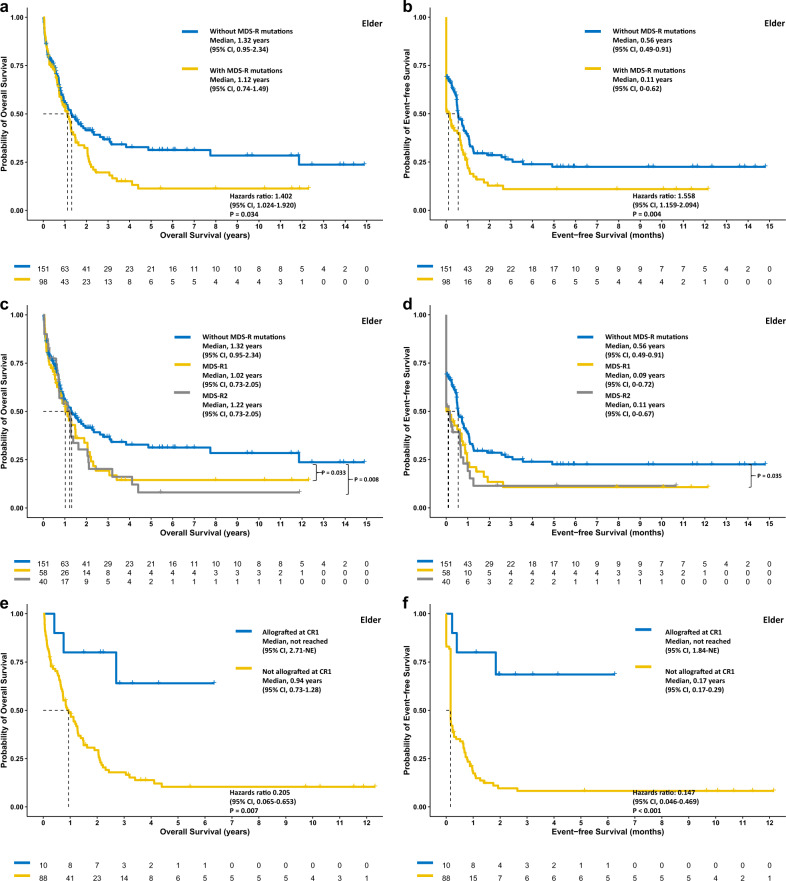
The Kaplan‒Meier survival curves for the elderly patient cohort. OS (**a**) and EFS (**b**) stratified by the status of MDS-R mutations, the MDS-R mutation burdens (**c** and **d**, respectively), and treatment with or without allogeneic transplantation at first remission (**e** and **f**, respectively). Patients with MDS-R mutations might benefit from allogeneic transplantation. MDS-R1, with one MDS-R mutation; MDS-R2, with 2 or more MDS-R mutations.

**Fig. 4:**
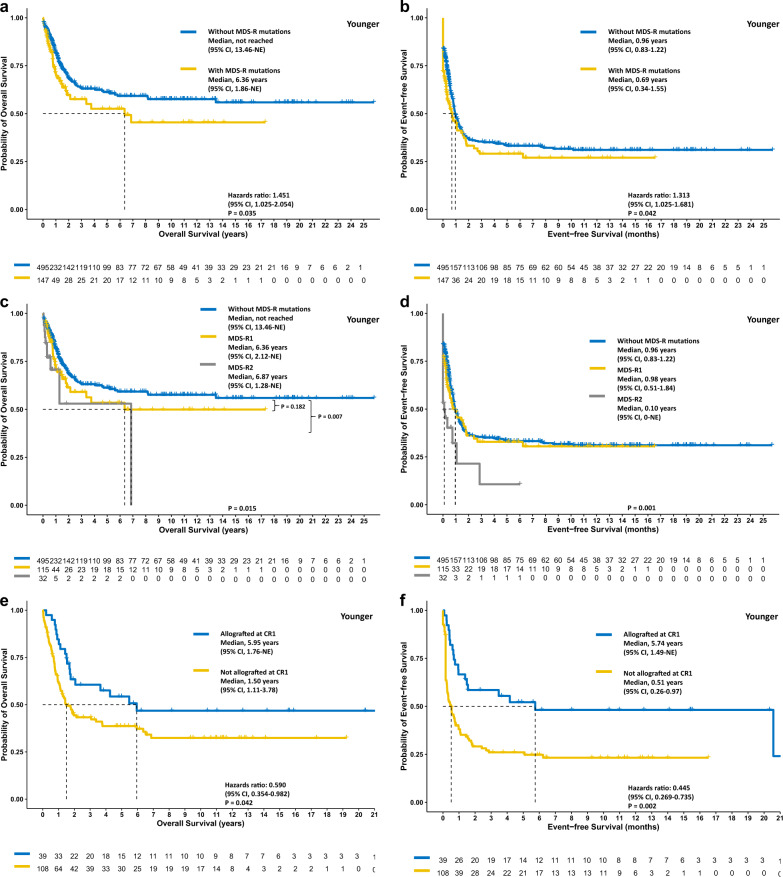
The Kaplan‒Meier survival curves for the younger patient cohort. OS (**a**) and EFS (**b**) of patients stratified by the status of MDS-R mutations, the MDS-R mutation burdens (**c** and **d**, respectively), and treatment with or without allogeneic transplantation at first remission (**e** and **f**, respectively). Patients receiving allogeneic transplantation at first remission experienced longer OS (**e**) and EFS (**f**). MDS-R1, with one MDS-R mutation; MDS-R2, with 2 or more MDS-R mutations.

In the total cohort, the older and the younger group, patients with MDS-R mutations had significantly poorer OS (*P* < 0.001, *P* = 0.034, and *P* = 0.035, respectively; Figs. 2a, 3a, and 4a) and EFS (*P* < 0.001, *P* = 0.004, *P* = 0.042, respectively; Figs. 2b, 3b, and 4b) but similar RFS (Supplementary Figs. 2–4). In the total cohort, patients with two or more MDS-R mutations experienced significantly poorer OS and EFS than those with only one MDS-R mutation or without MDS-R mutation (Fig. 2c, d); however in subgroup analyses, the same result was only observed for EFS in the younger group (Figs. 3c, d and 4c, d). In both age groups, patients with one MDS-R mutation shared similar dismal OS to those with two or more MDS-R mutations, compared with patients without MDS-R mutations (Figs. 3c and 4c).

While focusing on patients with intermediate-risk genotypes based on the 2017 ELN risk classification, MDS-R mutations were able to dichotomize the patients into two groups with distinct OS in the total cohort (Supplementary Fig. 5). Although patients with intermediate-risk genotypes/without MDS-R mutations had better OS than those with intermediate-risk genotypes/with MDS-R mutations (*P* < 0.001), their outcomes were still significantly poorer than those with favorable-risk genotypes (*P* = 0.006). Similarly, patients with intermediate-risk genotypes/with MDS-R mutations had significantly better outcomes than those with unfavorable-risk genotypes (*P* = 0.021). In the older patient group, the status of MDS-R mutations also effectively divided intermediate-risk patients into two groups with distinct outcomes (Supplementary Fig. 6). Conversely, in the younger patient group, intermediate-risk patients with MDS-R mutations shared similar OS to those without MDS-R mutations (Supplementary Fig. 7).

We included age, WBC count at diagnosis, disease risk based on the 2017 ELN classification, allo-HSCT at first CR, and MDS-R mutation status as covariables in multivariate Cox proportional hazards regression analysis model (Table 3). In addition to an older age, higher WBC count at diagnosis, and ELN intermediate- or adverse-risk, the presence of MDS-R mutations was also an independent unfavorable prognostic factor for both EFS (*P* = 0.040) and OS (*P* = 0.045), while receiving allo-HSCT at first CR was an independent favorable risk factor.

**Table 3:** Multivariate Cox proportional hazards regression analyses.

Variables	EFS				OS			
	HR	Lower	Upper	*P* value	HR	Lower	Upper	*P* value
Age^a^	1.011	1.006	1.016	<0.001	1.017	1.011	1.023	<0.001
WBC counts^a^ (k/μL)	1.002	1.001	1.003	<0.001	1.002	1.001	1.003	<0.001
2017 ELN classification^c^								
Intermediate vs. favorable	2.022	1.643	2.489	<0.001	2.257	1.792	2.843	<0.001
Adverse vs. favorable	3.809	3.060	4.740	<0.001	4.297	3.384	5.455	<0.001
HSCT at first CR^b^	0.317	0.250	0.402	<0.001	0.408	0.317	0.524	<0.001
MDS-R mutations	1.225	1.009	1.488	0.040	1.239	1.005	1.526	0.045

*CR* complete remission, *ELN* European LeukemiaNet, *MDS-R* myelodysplasia-related, *WBC* white blood cell.

^a^Continuous variables.

^b^HSCT at CR1 vs. HSCT at other disease statuses or without HSCT.

^c^Since AML with myelodysplasia-related gene mutations belongs to the adverse category in the 2022 ELN risk classification, we used the 2017, instead of 2022, ELN risk classification as a covariate.

### MDS-R mutations shape the response to chemotherapy

We hypothesized that patients with MDS-R mutations not only had a lower CR rate after induction chemotherapy but might also be prone to having residual leukemia cells even obtaining CR, which would lead to dismal outcomes. In the cohort of 291 patients who obtained CR and underwent serial MRD monitoring using NGS, 72 (24.7%) had MDS-R mutations at diagnosis; patients with MDS-R mutations more frequently required two cycles of induction chemotherapy to achieve CR than those without MDS-R mutations (37.5% vs. 17.4%, *P* = 0.001). Patients with MDS-R mutations had significantly higher odds of harboring NGS MRD at morphological CR after induction chemotherapy (63.9% vs. 40.6%, *P* = 0.001) and after the 1st consolidation chemotherapy (45.8% vs. 23.3%, *P* < 0.001) than patients without MDS-R mutations. Surprisingly, among patients with MDS-R mutations at diagnosis, all patients with MRD after induction therapy and 88% of those with MRD after the 1st consolidation had at least one MDS-R mutation at morphological CR, indicating the leukemia clones with MDS-R mutations might be relatively chemoresistant. Among the patients with MDS-R at diagnosis, the presence of NGS MRD predicted a higher early relapse rate (1-year cumulative incidence of relapse, 51.5% *vs*. 23.6%, *P* < 0.001), consistent with the concept that persistent MRD is predictive of early relapse.

### The impact of Allo-HSCT for patients with MDS-R mutations in different age groups

Considering the chemoresistance owing to MDS-R mutant clones, we speculated that receiving allo-HSCT at first CR might overcome the negative impact of MDS-R mutations. Overall, the proportions of patients receiving allo-HSCT at first remission were comparable between patients with and without MDS-R mutations (*P* = 0.755 for the younger group and 0.447 for the older group, respectively). In the total cohort, landmark analysis revealed that patients with MDS-R mutations had significantly better OS and EFS if they underwent allograft at first remission (both *P* < 0.001, Fig. 2e, f). Subgroup analyses showed similar results for both the older group (*P* = 0.007 and <0.001, respectively, Fig. 3e, f) and the younger group (*P* = 0.042 and 0.002, respectively, Fig. 4e, f). Notably, as only a limited number of patients (*n* = 10) with MDS-R mutations in the older group underwent allografting, it is necessary to validate this finding in a larger patient cohort.

## Discussion

In recent years, AML classification has changed from morphological discrimination alone to incorporation of aberrations detected using genomic and transcriptomic-based systems. Recently, Lindsley et al. defined a set of eight gene mutations described above, as sAML-type mutations owing to their strong association with secondary AML transformed from MDS and CMMoL in the analysis of a cohort of 194 patients. The authors found that elderly *de novo* AML patients carrying sAML-type mutations shared similar clinicopathological features with clinically confirmed secondary AML patients and validated the findings in another cohort of 105 elderly patients [7]. In addition, Gardin et al. evaluated sAML-type mutations in a cohort of 509 patients aged 60 years or older with *de novo*, secondary, or therapy-related AML, and found that sAML-type mutations might provide additional prognostic information for elderly patients with intermediate-risk genotypes defined by the 2017 ELN recommendation [8]. Based on these findings, the 2022 ICC categorized AML with these eight sAML-type mutations together with *RUNX1* mutation, which is also closely associated with sAML [7, 8] and confers poor prognosis [23], as AML with MDS-R gene mutations [9]. Though the poor impact of MDS-R mutations on elderly AML patients with *de novo* AML is clear, the clinical significance of these mutations in younger patients remains unclear. To the best of our knowledge, this study is the first to show the adverse effect of MDS-R mutations on clinical outcomes of younger patients with *de novo* AML. In addition, this study also comprehensively elucidated the correlation of MDS-R mutations with clinical features and their interaction with other gene mutations in both younger and older age groups in a large *de novo* AML cohort comprising 56% of younger patients.

We found that MDS-R mutations had significantly negative prognostic impacts on OS and EFS of the younger patients, similar to the older patients. Nevertheless, incorporating MDS-R mutations did not further dichotomize the 2017 ELN-defined intermediate-risk patients in the younger age group while they could in the older one. Two explanations for this phenomenon are proposed. First, most patients carrying MDS-R mutations harbored *ASXL1* (40.6%) or *RUNX1* (41.6%) mutations, and thus they would be included in the adverse-risk group defined by the 2017 ELN classification. While focusing on intermediate-risk patients, a group that excluded patients with *ASXL1* or *RUNX1* mutations, only 14.3% of younger patients had MDS-R mutations, as compared with 32.2% of older patients. The very limited patient number might compromise the statistical power. Second, among patients with intermediate-risk genotypes, *FLT3*-ITD^high allelic ratio^ was significantly more prevalent among younger patients without MDS-R mutations than among those with MDS-R mutations (*P* = 0.033, Supplementary Table 2). Although all these patients had concurrent *NPM1* mutations, their prognosis might still be worse than that of patients with wild-type *NPM1* and *FLT3*, as reported previously [24, 25]. Furthermore, because of the enrollment timeframe, many patients with *FLT3-*ITD in our cohort did not receive FLT3 inhibitor treatment upfront, which also compromised their outcomes.

Though the 2022 ICC defined AML with MDS-R gene mutations exclude those with *TP53* mutations [9, 10], we included all patients with MDS-R mutations regardless of the presence or absence of *TP53* mutations since the aim of this study was to evaluate the clinical relevance and prognostic impact of MDS-R mutations in AML as that was done in the study of Lindsley et al. [7]. Indeed, the patient cohort of MDS-R mutations in this study included a few patients with ICC and ELN-2022 defined AML with mutated *TP53*. In the analysis focusing only on patients without *TP53* mutations, we found that the poor prognostic impact of MDS-R mutations remained: the patients with MDS-R mutations had poorer OS (*P* < 0.001 for total cohort; *P* = 0.006 for elderly patients; *P* = 0.038 for younger patients) compared with those without MDS-R mutations.

In this study, 23.4% of younger patients and 44.9% of elderly patients harbored MDS-R mutations. The percentage of elderly patients with MDS-R mutations in our cohort was similar to that of the rigorously clinically defined *de novo* AML cohort reported by Lindsley et al. (33.3%, *P* = 0.147) and Gardin et al. (42.9%, *P* = 0.572) [7, 8]. These results indicate that a significant portion of the patients with *de novo* AML carry MDS-R mutations, although they do not have a history of cytopenia or any hematologic disease. The higher percentages of MDS-R mutations in elderly patients may partially explain why these groups of patients, even those receiving standard chemotherapy, have significantly poorer outcomes than younger patients [2]. It is interesting that although the presence of MDS-R mutations could discriminated a group of *de novo* AML patients whose outcomes resembled those of patients with secondary AML, MDS-R mutations did not correlate with the presence of myelodysplasia-related cytogenetic changes or adverse-risk cytogenetics, consistent with the previous report [8]. This result emphasizes the necessity of performing comprehensive molecular studies for patients with newly diagnosed AML to concisely identify those at high risk.

According to Gardin et al., allo-HSCT might overcome the negative impact of MDS-R mutations on elderly patients [8]. In the present study, allo-HSCT provided survival benefits for both younger and older patients carrying MDS-R mutations. In recent years, with advances in transplant strategies and graft-versus-host-disease prophylaxis and treatment, an increasing number of patients can receive allo-HSCT from alternative donors. However, less than 20% of elderly patients receive allografts at first remission [26, 27]. In general, exploring the optimal treatment strategy for elderly patients with MDS-R mutations who are not suitable for transplantation is warranted.

In conclusion, AML patients with MDS-R mutations have distinct clinical features and poor outcomes regardless of older or younger age. Allo-HSCT might improve the prognosis of AML patients carrying MDS-R gene mutations.

## Supplementary information

Supplementary

## Data Availability

The datasets generated during and/or analyzed during the current study are available from the corresponding author on reasonable request.
