# Supplementary material for: Poor prognostic implications of myelodysplasia-related mutations in both older and younger patients with de novo AML
Source: Blood Cancer J. 2023 Jan 4;13(1):4. doi: 10.1038/s41408-022-00774-7 (PMC9813374; doi:10.1038/s41408-022-00774-7)
Supplement: Supplementary file 1 — Supplementary [file 41408_2022_774_MOESM1_ESM.pdf]

Poor prognostic implications of myelodysplasia-related gene mutations in both older and younger patients with *de novo* acute myeloid leukemia

Supplementary Table 2
Supplementary Figure Legend 5
Supplementary Figures 7

Supplementary Table 1.
Full list of genes studied in targeted NGS sequencing

Gene Name	Target region (exon)	Gene Name	Target region (exon)
ABL	4-6	*KDM6A*	full
ASXL1	12	*KIT*	2, 8-11, 13+17
ATRX	8-10 and 17-31	*KRAS*	2+3
BCOR	full	*MLL*	5-8
BCORL1	full	*MPL*	10
BRAF	15	*MYD88*	3-5
CALR	9	*NOTCH1*	26-28, 34
CBL	8+9	*NPM1*	12
CBLB	9, 10	*NRAS*	2+3
CBLC	9, 10	*PDGFRA*	12, 14,18
CDKN2A	full	*PHF6*	full
CEBPA	full	*PTEN*	5+7
CSF3R	14-17	*PTPN11*	3+13
CUX1	full	*RAD21*	full
DNMT3A	full	*RUNX1*	full
ETV6	full	*SETBP1*	4(partial)
EZH2	full	*SF3B1*	13-16
FBXW7	9+10+11	*SMC1A*	2, 11, 16+17
FLT3	14+15+20	*SMC3*	10, 13, 19, 23, 25+28
GATA1	2	*SRSF2*	1
GATA2	2-6	*STAG2*	full
GNAS	8+9	*TET2*	3-11
HRAS	2+3	*TP53*	2-11
IDH1	4	*U2AF1*	2+6
IDH2	4	*WT1*	7+9
IKZF1	full	*ZRSR2*	full
JAK2	12+14	*KDM6A*	full
JAK3	13	*KIT*	2, 8-11, 13+17

Supplementary Table 2.
Comparison of gene mutations between intermediate-risk patients with and without MDS-R mutations

Categories	Variables	Int-risk cohort (n = 317)	Younger population			Older population		
			Without MDS-R mutations (n = 168, 85.7%)	With MDS-R mutations (n = 28, 14.3%)	P value	Without MDS-R mutations (n = 82, 67.8%)	With MDS-R mutations (n = 39, 32.2%)	P value
Activated signaling	*FLT3*-ITD^{High} [αβ]	68 (21.5%)	36 (21.4%)	1 (3.6%)	0.033	24 (29.3%)	7 (17.9%)	0.265
	FLT3-ITD^{Low} [αβ]	23 (7.3%)	20 (11.9%)	2 (7.1%)	0.746	1 (1.2%)	0 (0%)	>0.999
	FLT3-TKD	19 (6.0%)	10 (6.0%)	2 (7.1%)	0.683	6 (7.3%)	1 (2.6%)	0.427
	KIT	10 (3.2%)	4 (2.4%)	1 (3.6%)	0.541	5 (6.1%)	0 (0%)	0.174
	NRAS	42 (13.2%)	25 (14.9%)	5 (17.9%)	0.776	9 (11.0%)	3 (7.7%)	0.750
	KRAS	12 (3.8%)	9 (5.4%)	1 (3.6%)	>0.999	1 (1.2%)	1 (2.6%)	0.543
	PTPN11	18 (5.7%)	10 (6.0%)	2 (7.1%)	0.683	4 (4.9%)	2 (5.1%)	>0.999
Tumor suppressor	*WTI*	31 (9.8%)	21 (12.5%)	2 (7.1%)	0.540	6 (7.3%)	2 (5.1%)	>0.999
	PHF6	11 (3.5%)	4 (2.4%)	3 (10.7%)	0.062	2 (2.4%)	2 (5.1%)	0.593
	NPM1	68 (21.5%)	36 (21.4%)	1 (3.6%)	0.033	24 (29.3%)	7 (17.9%)	0.265
DNA methylation	*IDH1*	29 (9.1%)	14 (8.3%)	2 (7.1%)	>0.999	8 (9.8%)	5 (12.8%)	0.754
	IDH2	48 (15.1%)	14 (8.3%)	9 (32.1%)	0.001	14 (17.1%)	11 (28.2%)	0.229
	DNMT3A	80 (25.2%)	38 (22.6%)	7 (25.0%)	0.809	27 (32.9%)	8 (20.5%)	0.200
	TET2	41 (12.9%)	12 (7.1%)	0 (0%)	0.222	19 (23.2%)	10 (25.6%)	0.821
Cohesin complex genes	*STAG2*	18 (5.7%)	0 (0%)	8 (28.6%)	<0.001	0 (0%)	10 (25.6%)	<0.001
	RAD21	5 (1.6%)	2 (1.2%)	0 (0%)	>0.999	1 (1.2%)	2 (5.1%)	0.243
	SMC1A	11 (3.5%)	5 (3.0%)	1 (3.6%)	>0.999	4 (4.9%)	1 (2.6%)	>0.999
	SMC3	2 (0.6%)	1 (0.6%)	0 (0%)	>0.999	1 (1.2%)	0 (0%)	>0.999
Transcription factor	*GATA2*	19 (6.0%)	10 (6.0%)	3 (10.7%)	0.404	5 (6.1%)	1 (2.6%)	0.663
	ETV6	3 (0.9%)	3 (1.8%)	0 (0%)	>0.999	0 (%)	0 (%)	>0.999

Chromatin modifiers	*BOCR*	8 (2.5%)	0 (0%)	4 (14.3%)	<0.001	0 (0%)	4 (10.3%)	0.010
	EZH2	3 (0.9%)	0 (0%)	2 (7.1%)	0.020	0 (0%)	1 (2.6%)	0.322
Spliceosome complex genes	*SF3B1*	5 (1.6%)	0 (0%)	2 (7.1%)	0.020	0 (0%)	3 (7.7%)	0.032
	SRSF2	19 (6.0%)	0 (0%)	3 (10.7%)	0.023	0 (0%)	16 (41.0%)	<0.001
	U2AF1	18 (5.7%)	0 (0%)	11 (39.3%)	<0.001	0 (0%)	7 (17.9%)	<0.001
	ZRSR2	3 (0.9%)	0 (0%)	1 (3.6%)	0.143	0 (0%)	2 (5.1%)	0.102

[α] One young patients with *FLT3*-ITD did not have allelic ratio data. This patient had no MDS-R mutations.

[β] Defined as *FLT3* mutated/wild-type allelic ratio ≥ 0.5.

Supplementary Figure 1. Flow chart of patient selection

Supplementary Figure 2. The relapse-free survival stratified by the status of MDS-R mutations among the total cohort**.**

Supplementary Figure 3. The relapse-free survival stratified by the status of MDS-R mutations among the elder patients**.**

Supplementary Figure 4. The relapse-free survival stratified by the status of MDS-R mutations among the younger patients**.**

Supplementary Figure 5. The overall survival stratified by the ELN 2017 classification among the total cohort. The intermediate-risk group can be well dichotomized by the status of MDS-R mutations. Red line: patients with favorable-risk genotypes; Yellow line: patients with intermediate-risk genotypes and without MDS-R mutations; Gray line: patients with intermediate-risk genotypes and with MDS-R mutations; Red line: patients with unfavorable-risk genotypes.

Supplementary Figure 6. The overall survival stratified by the ELN 2017 classification among the elder patients. The intermediate-risk group can be dichotomized by the status of MDS-R mutations. Red line: patients with favorable-risk genotypes; Yellow line: patients with intermediate-risk genotypes and without MDS-R mutations; Gray line: patients with intermediate-risk genotypes and with MDS-R mutations; Red line: patients with unfavorable-risk genotypes.

Supplementary Figure 7. The overall survival stratified by the ELN 2017 classification among the younger patients. Among the patients with intermediate-risk genotypes, those with MDS-R mutations had similar survival to those without MDS-R mutations. Red line: patients with favorable-risk genotypes; Yellow line: patients with intermediate-risk genotypes and without MDS-R mutations; Gray line: patients with

intermediate-risk genotypes and with MDS-R mutations; Red line: patients with unfavorable-risk genotypes.

Supp Figure 1

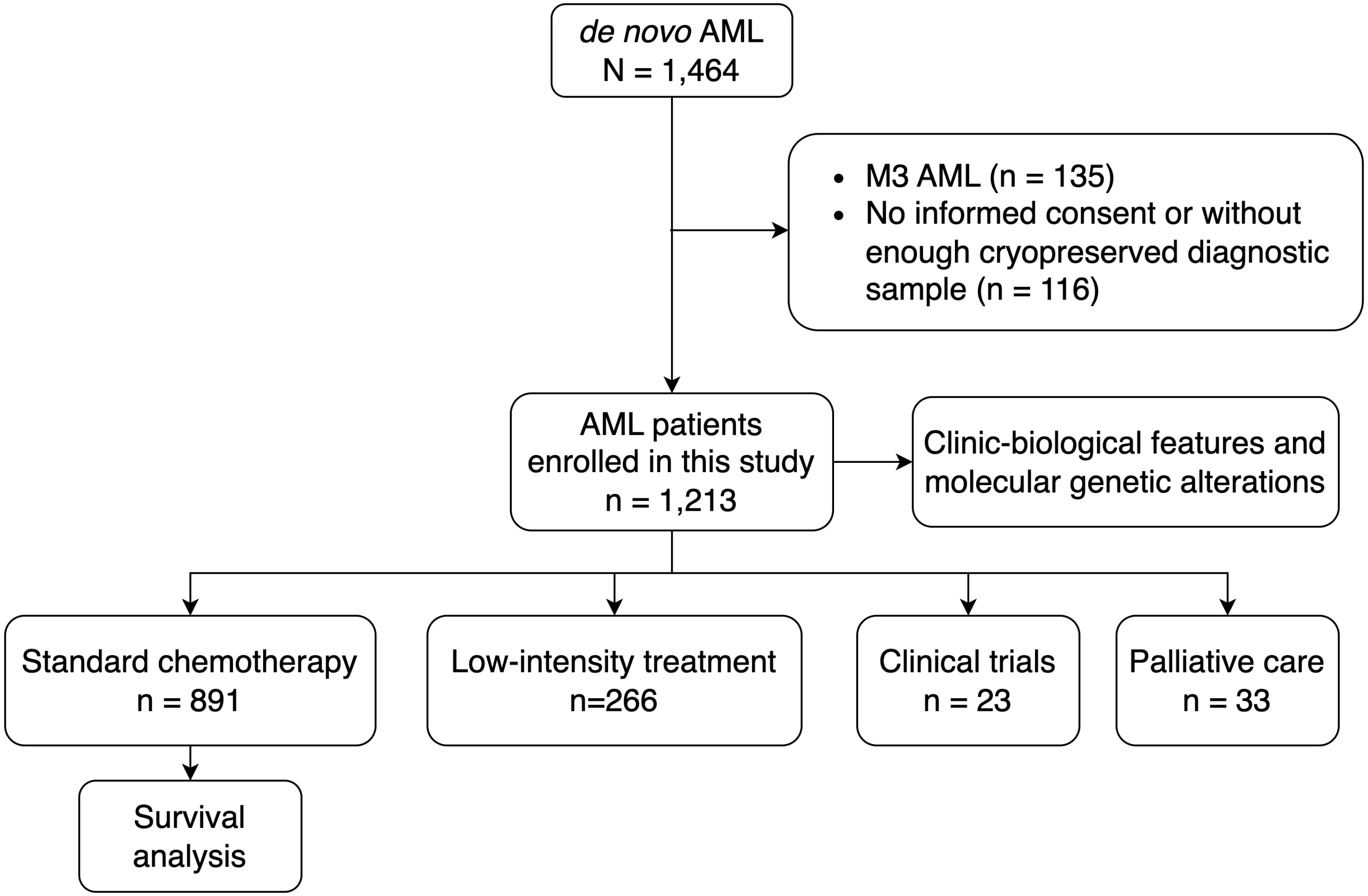

Supp Figure 2
Total cohort

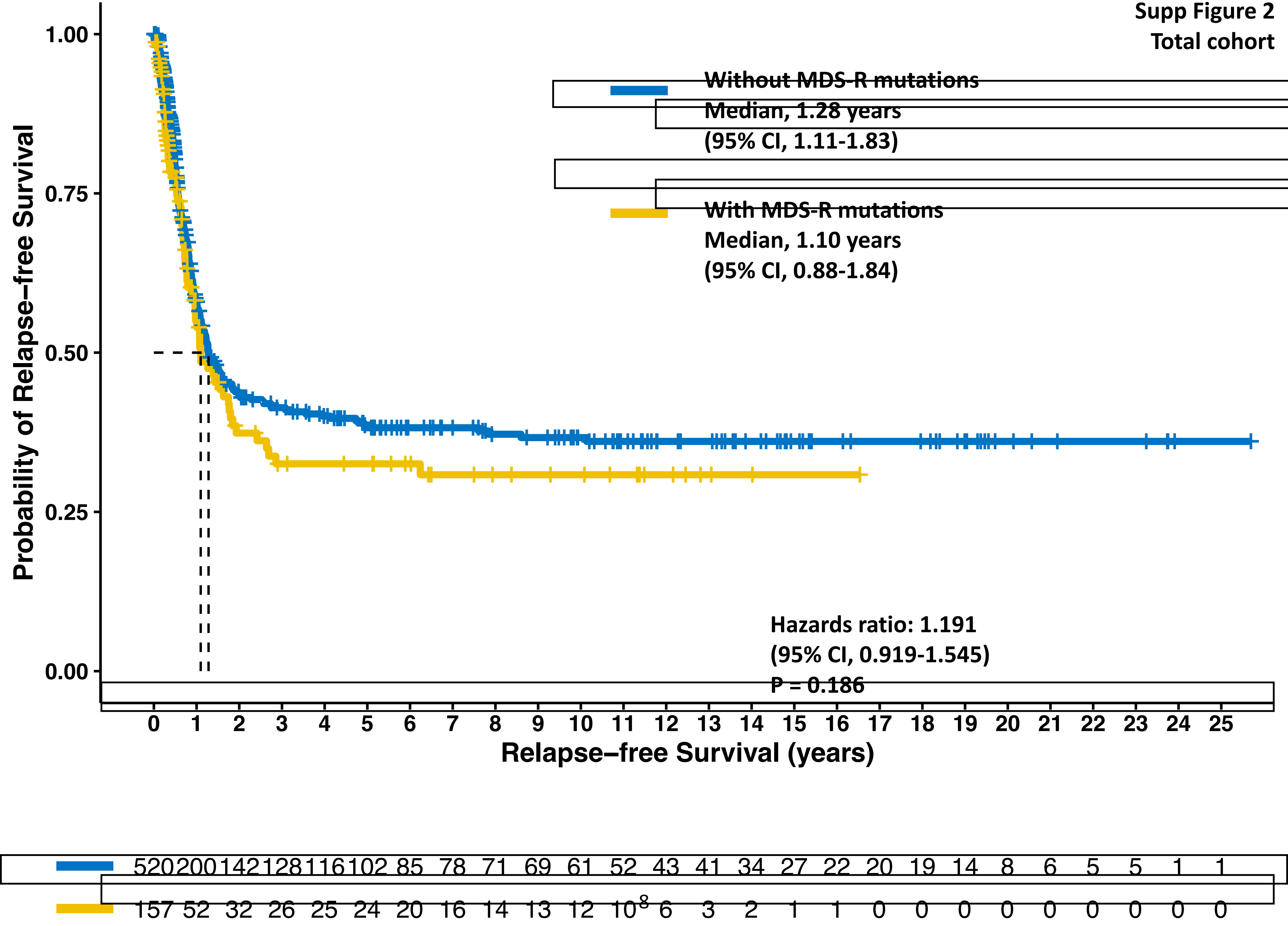

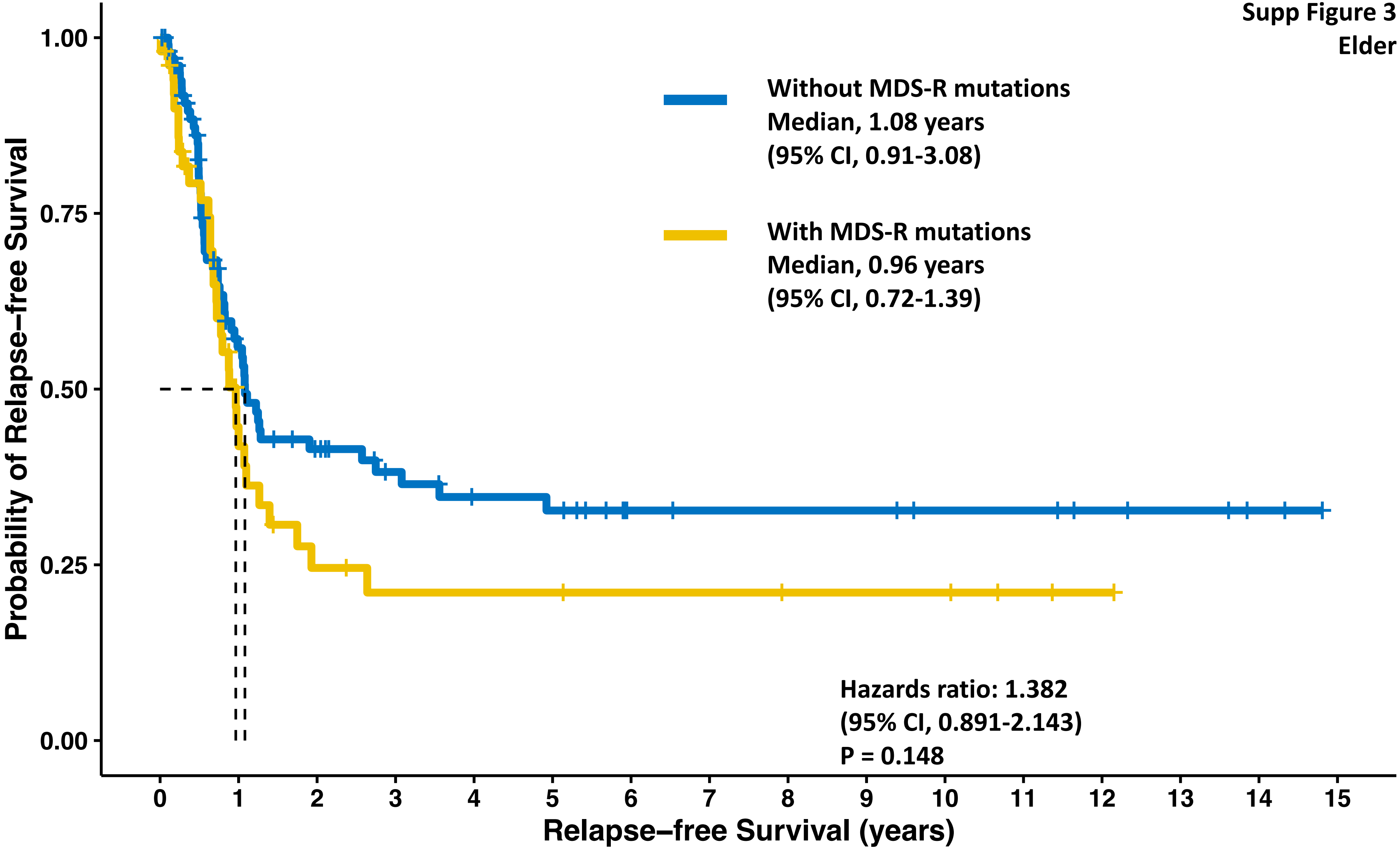

Without MDS-R mutations	104	43	29	22	18	17	10	9	9	9	7	7	5	4	2	0
With MDS-R mutations	51	16	8	6	6	6	5	5	4	4	4	2	1	0	0	0

Supp Figure 4
Younger

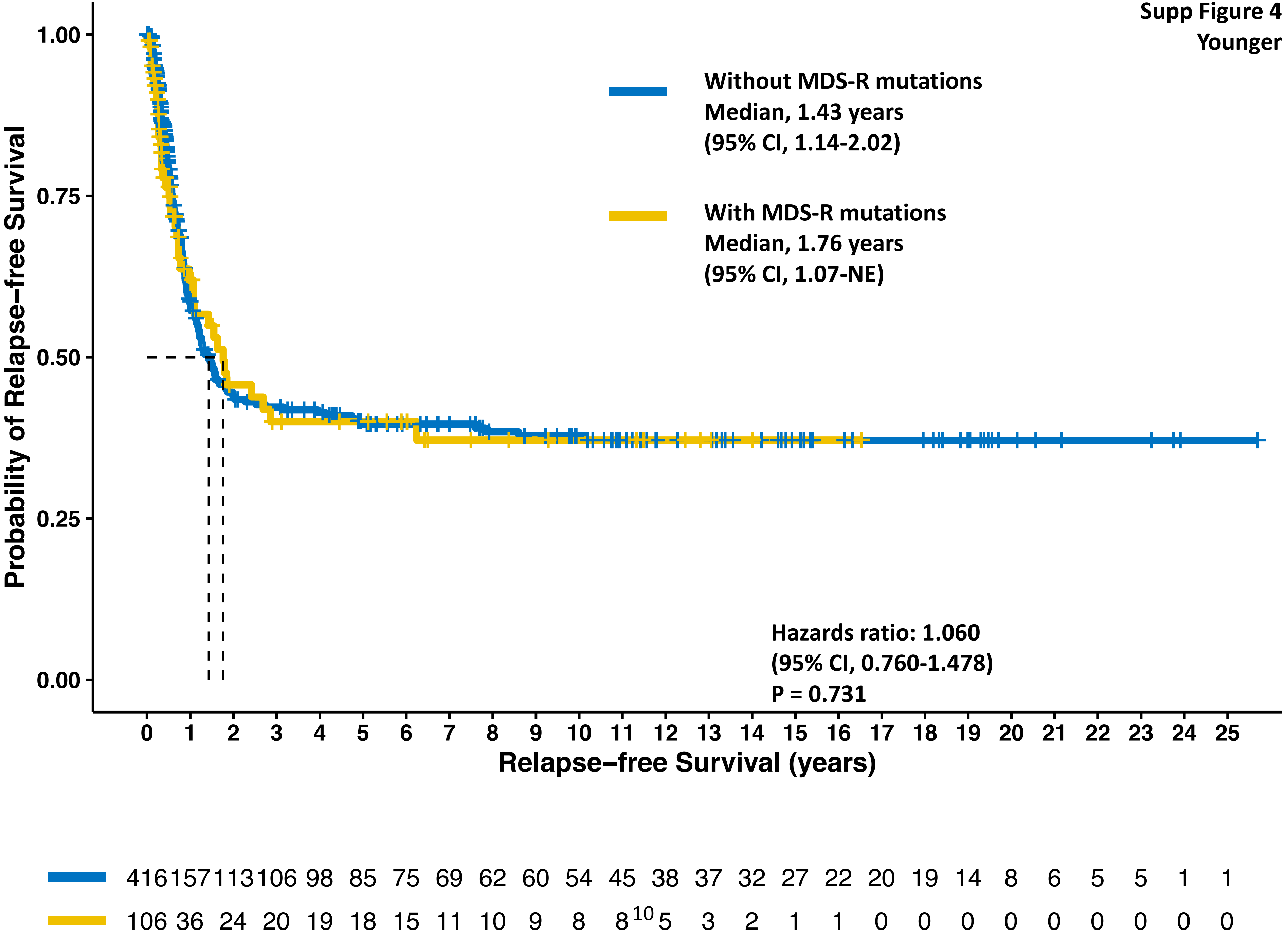

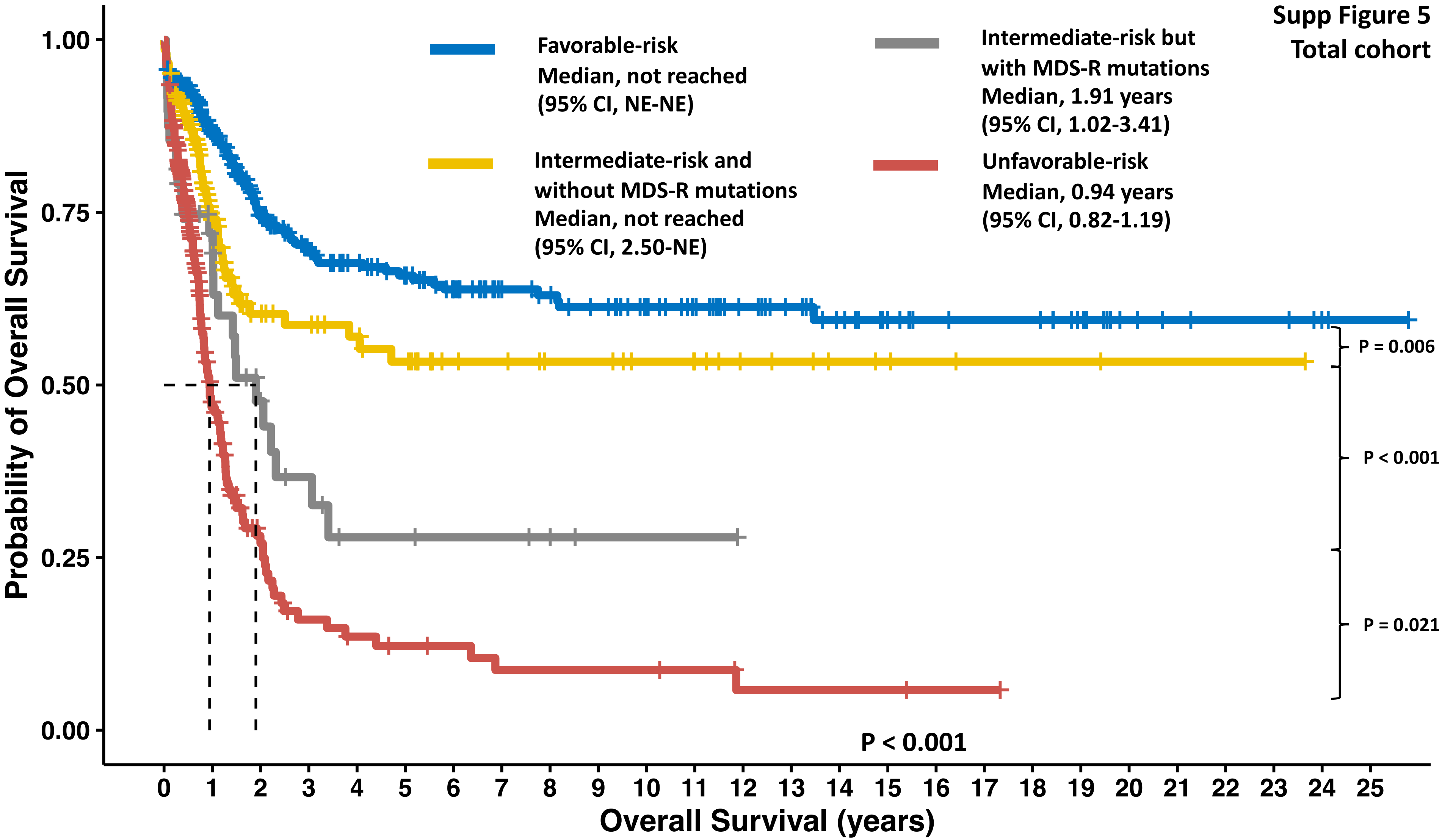

Favorable-risk (blue)	347	221	155	127	114	104	90	77	74	69	60	52	42	37	30	24	20	19	19	14	8	6	5	5	2	1
Intermediate-risk without MDS-R (yellow)	206	78	41	37	33	29	20	19	16	16	13	11	8	7	5	4	3	2	2	2	1	1	1	1	0	0
Intermediate-risk with MDS-R (grey)	48	25	13	9	5	5	4	4	2	1	1	1	0	0	0	0	0	0	0	0	0	0	0	0	0	0
Unfavorable-risk (red)	290	63	25	13	10	8	7	5	5	5	5	4	2	2	2	2	1	1	0	0	0	0	0	0	0	0

Supp Figure 6
Elder

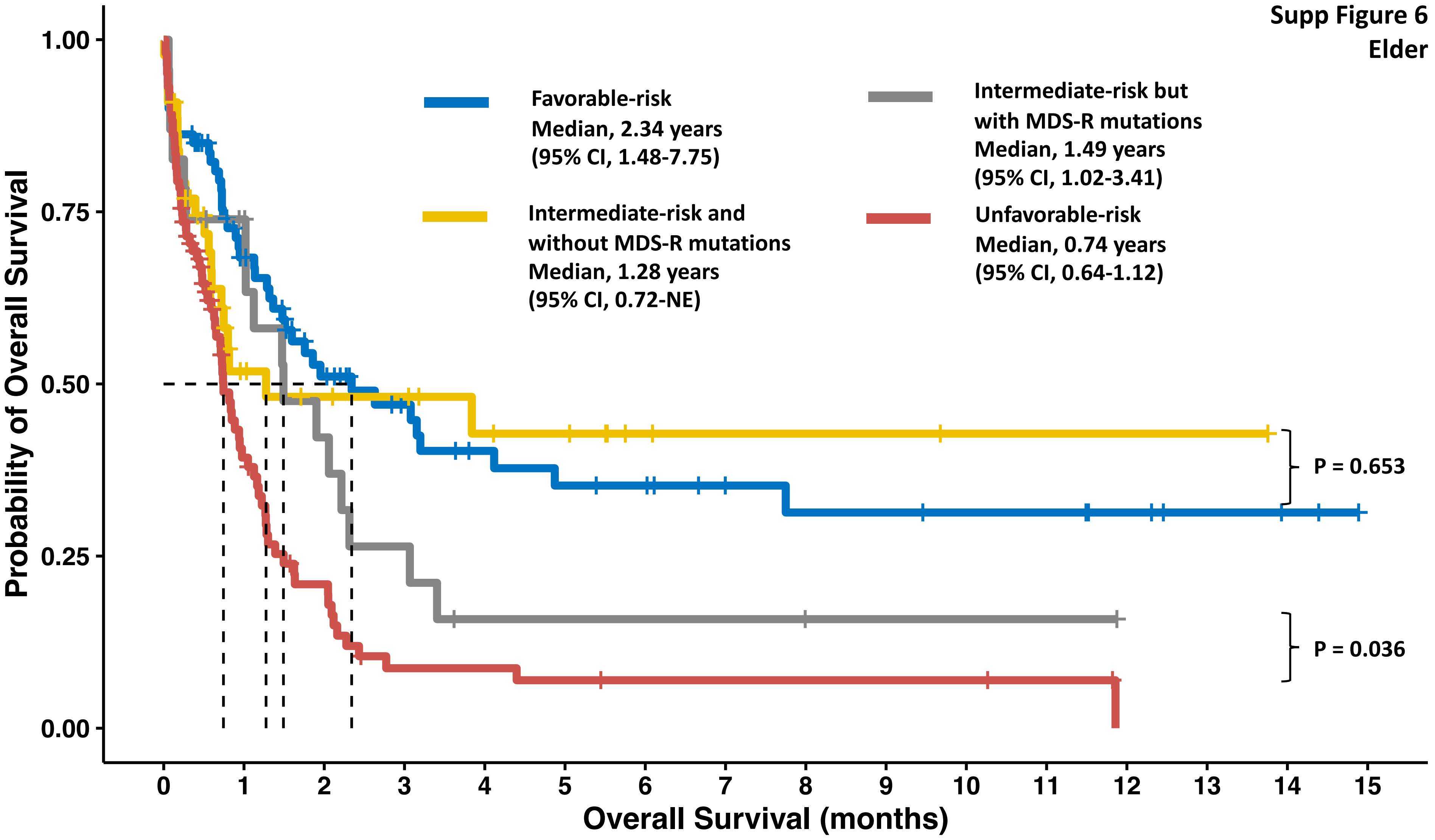

	0	1	2	3	4	5	6	7	8	9	10	11	12	13	14	15
(Blue)	80	47	30	21	16	14	13	9	8	8	7	7	5	3	2	0
(Yellow)	44	15	12	11	8	7	3	2	2	2	1	1	1	1	0	0
(Grey)	23	15	8	5	2	2	2	2	1	1	1	1	0	0	0	0
(Red)	102	29	14	5	5	4	3	3	3	3	3	2	0	0	0	0

Supp Figure 7
Younger

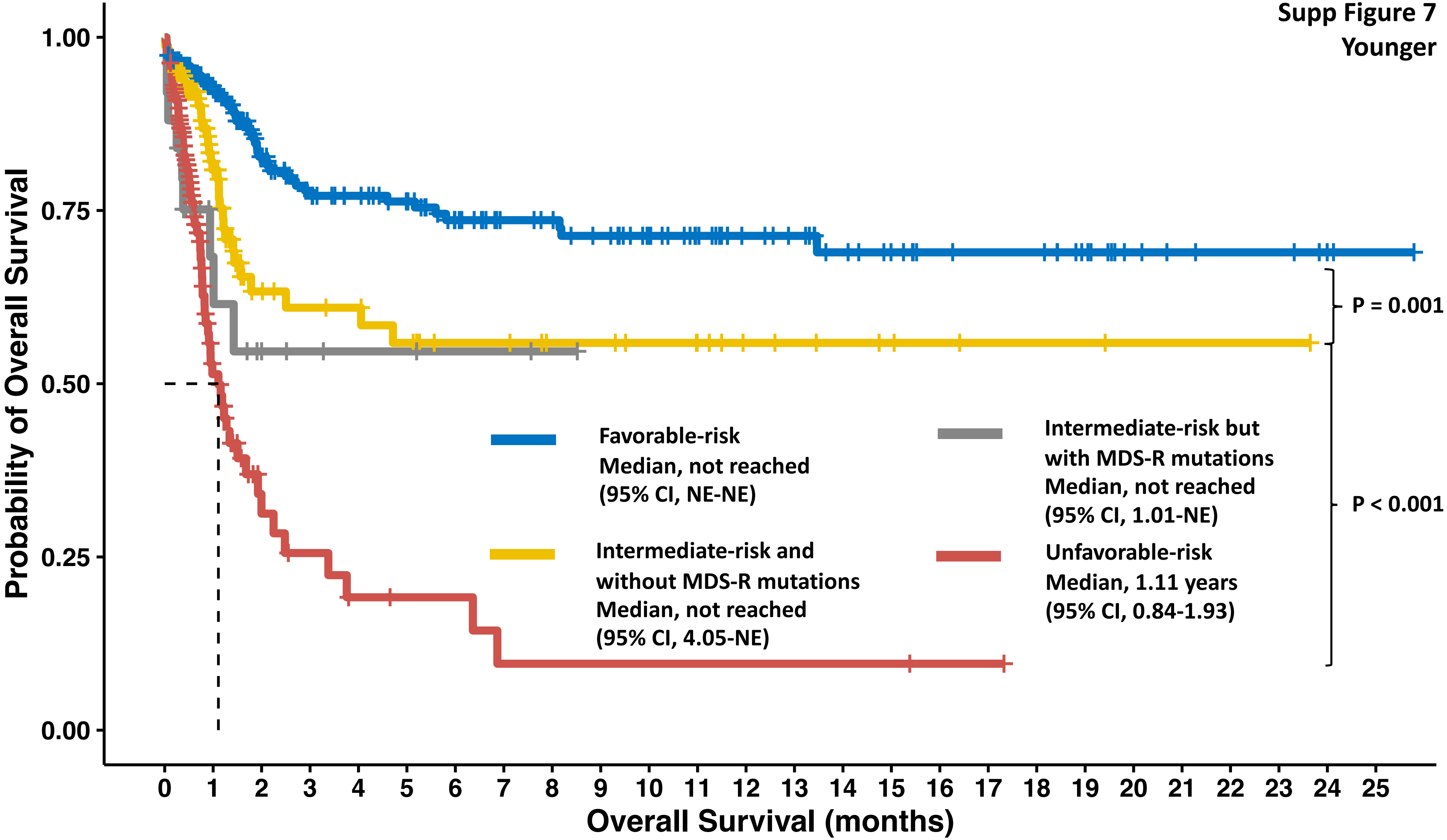

267	174	125	106	98	90	77	68	66	61	53	45	37	34	28	24	20	19	19	14	8	6	5	5	2	1
162	63	29	26	25	22	17	17	14	14	12	10	7	6	5	4	3	2	2	2	1	1	1	1	0	0
25	10	5	4	3	3	2	2	1	0	0	0	0	0	0	0	0	0	0	0	0	0	0	0	0	0
188	34	11	8	5	4	4	2	2	2	2	2	2	2	2	2	1	1	0	0	0	0	0	0	0	0
